# Meiotic Development of Canine Oocytes from Poly-Ovular and Mono-Ovular Follicles after In Vitro Maturation

**DOI:** 10.3390/ani13040648

**Published:** 2023-02-13

**Authors:** Igor Astudillo, Karla Aspee, Jaime Palomino, Oscar A. Peralta, Victor H. Parraguez, Monica De los Reyes

**Affiliations:** 1 Laboratory of Animal Reproduction, Department of Animal Production, Faculty of Veterinary Sciences, University of Chile, Santiago 8820000, Chile; 2 College of Veterinary Medicine, Faculty of Medical Sciences, Bernardo O’Higgins University, Santiago 8320000, Chile; 3 Laboratory of Animal Physiology, Department of Biological Sciences, Faculty of Veterinary Sciences, University of Chile, Santiago 8820000, Chile

**Keywords:** dogs, in vitro, maturation, follicle, meiosis

## Abstract

**Simple Summary:**

Mammalian ovaries contain many oocytes that are enclosed in different follicles, commonly one oocyte in one follicle. Poly-ovular follicles are those with more than one oocyte within a single follicle. This type of follicle has been observed in many mammals, with an especially high incidence in canines, representing an intriguing condition. The quality of oocytes from poly-ovular follicles is heterogeneous, as different oocyte morphologies have been observed. Oocyte meiotic potential has been a key factor in determining the success rate of the in vitro maturation process in canines because of the mixed population of oocytes collected from follicles at different stages of development and at random phases of the estrus cycle. Several oocytes may be derived from poly-ovular follicles without knowledge of their meiotic capacity in vitro. Therefore, to improve in vitro maturation and reproductive biotechnologies in canines, evaluating the maturity potential of oocytes according to follicle type and estrous cycle is necessary. Our study demonstrated that the efficiency of in vitro maturation of oocytes retrieved from poly-ovular or mono-ovular follicles depends on the follicle type according to the phases of the estrous cycle during collection, which is important to consider when improving in vitro maturation protocols in this species.

**Abstract:**

Poly-ovular follicles are defined as those with more than one oocyte present in single follicles. The occurrence frequency of this follicle type is higher in canines than that in other species. This study aimed to evaluate the in vitro meiotic maturation of dog oocytes from this follicle type in comparison to those from mono-ovular follicles of various sizes (small antral, medium antral, and large antral) considering different phases of the estrus cycle (anestrus, proestrus, estrus, and diestrus). Canine oocytes were obtained separately from the poly-ovular and mono-ovular antral follicles from the ovaries of adult females. In each experimental replicate, cumulus-oocyte complexes (COCs) from poly-ovular and mono-ovular follicles were incubated in supplemented TCM-199 at 38.5 °C and 5% CO_2_ for 72 h. After culturing, the meiotic development of each oocyte was evaluated using epifluorescence microscopy. Meiotic stages were classified into germinal vesicle (GV), germinal vesicle breakdown (GVBD), first metaphase (MI), and second metaphase (MII). Data were evaluated using an analysis of variance. Oocytes from poly-ovular follicles at all phases exhibited a higher (*p* < 0.05) percentage of oocytes arrested at the GV stage than those from mono-ovular follicles, showing the highest rate of GV in small antral follicles during anestrus. In contrast, there were no differences in MII rates (*p* < 0.05) in oocytes from mono-ovular and poly-ovular follicles during the estrus and diestrus phases in all sizes evaluated, with the highest MII rate in estrus. These results suggest that oocytes from poly-ovular follicles can resume meiosis at a slower rate than those from mono-ovular follicles; however, the maturation in vitro of such oocytes is possible. Furthermore, the relationship between the maturation capacity of oocytes from both poly-ovular and mono-ovular follicles depends on the ovarian cycle and follicular development.

## 1. Introduction

Poly-ovular follicles contain more than one oocyte per follicle. Although infrequently occurring in most mammals, this type of follicle has a high incidence in the ovaries of canine species [1,2,3].

During follicular development, the oogonia show intercellular cytoplasmic bridges that form nests during growth in the developing ovary [4]. Nest breakdown occurs because of invasion into the oogonia nests by pre-granulosa cells [5]. Primordial follicles develop when the nests undergo programmed breakdown [6]. The timing of nest breakdown and primordial follicle formation varies among species [7]. Canine ovaries contain only nests of oogonia at birth, and the first primordial follicles are observed approximately three weeks later [8]. Therefore, the genesis of poly-ovular follicles could be because the oogonia nests could not be separated at birth, causing the development of follicles with an incomplete breakage of cytoplasmic connecting germ cells during the breakdown, giving rise to poly-ovular follicles [9]. However, evidence supporting this mechanism is mainly derived from the interpretation of data from knockout models. Failure to separate germ cells during the early phases of folliculogenesis [10,11] and fusion of adjacent individual follicles have also been proposed as alternative mechanisms that could explain the formation of poly-ovular follicles [12].

The significance of these follicles in canine reproduction is important due to the high number of occurrences. According to a previous study, oocytes in poly-ovular follicles start their development at a similar size to those in mono-ovular follicles, but differences appear later, suggesting some developmental lag in oocytes from poly-ovular follicles [10].

Morphological analysis has revealed poly-ovular follicles containing oocytes that are discordant in maturity [11]. Thus, oocytes can develop at different rates even though they are exposed to a similar follicular environment. Therefore, oocyte quality may be heterogeneous [4]. In addition, asynchronous maturation of oocytes enclosed within the same follicle has been observed in human ovaries [13], but without differences in the ability to be fertilized [14].

To date, the meiotic competence of dog oocytes from poly-ovular follicles remains unknown. The in vitro maturation (IVM) process depends greatly on the prior period inside the follicles under the influence of intraovarian factors and hormonal conditions. The canine oocytes used for maturation in culture protocols are usually obtained by the mincing method (cutting the ovary into small pieces). Although mincing of ovarian tissue may yield high numbers of oocytes, the population of oocytes recovered in this way are at random stages of development, regardless of the follicular condition (mono- or poly-ovular). Since oocyte meiotic development is a limiting factor for IVM efficiency in canines, many oocytes used for IVM may be derived from poly-ovular follicles; however, nothing is known about the ability of these oocytes to mature in culture. Therefore, this study aimed to evaluate the meiotic maturation of dog oocytes from poly-ovular follicles compared to those from mono-ovular follicles, specifically examining the follicle development stage and the estrus cycle.

## 2. Materials and Methods

### 2.1. Ovaries Processing and Oocyte Collection

Ovaries were obtained from mixed-breed non-pregnant female dogs between 1 to 4 years of age that underwent elective ovariohysterectomy at local veterinary clinics near our laboratory. The ovaries were placed into saline solution (0.9%, *w/v* NaCl) at 37 °C with 100 IU/mL penicillin G and 0.1% streptomycin sulfate (Merck KGaA, Darmstadt, Germany). All samples were transported to the laboratory within 20–30 min.

At the laboratory, each ovary was washed in phosphate-buffered saline (PBS; 137 mM NaCl, 2.7 mM KCl, 4.3 mM Na2HPO4, and 1.47 mM KH2PO4; pH 7.4). Individual antral follicles from ovaries at different phases of the estrus cycle—anestrus (*n* = 16), proestrus (*n* = 11), estrus (*n* = 17), and diestrus (*n* = 31)—were dissected free of ovarian tissue under a stereomicroscope (Meiji Techno SKT, Tokyo, Japan).

The phases of the estrus cycle of each donor were assessed by evaluating the presence or absence of follicles and corpus luteum (CL) on the ovarian surface [15] and confirmed by progesterone analysis, according to our previous studies [16,17]. In brief, anestrus was defined as <0.2 ng/mL progesterone and absence of follicles or CL on the ovarian surface; proestrus as 0.2–1.9 ng/mL progesterone and small to medium follicles growing on the ovarian surface; estrus as 2–19 ng/mL progesterone and medium and large follicles on the ovarian surface; and diestrus as >20 ng/mL progesterone and mainly CLs on the ovaries with some small and medium follicles. Plasma progesterone concentrations were evaluated using an enzyme-linked immunosorbent assay (Phomo Microplate Reader^®^; Autobio Labtec Instruments, Zhenghaidong, China) with a progesterone canine kit (MyBioSource^®^; San Diego, CA, USA). The sensitivity of the assay was 0.19 ng/mL.

**Figure 1:**
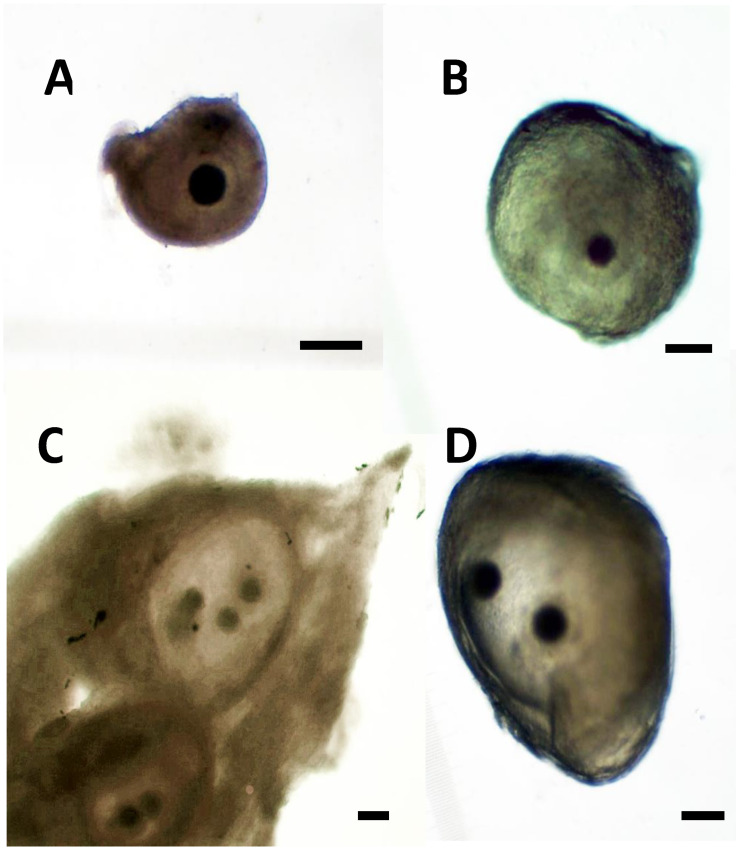
Representative photographs of (**A**,**B**) mono-ovular follicles with just one oocyte inside and (**C**,**D**) poly-ovular antral follicles with two or more oocytes inside each follicle. Bar = 100 μm, 40× magnification.

After careful dissection of the follicles, the cumulus–oocyte complexes (COCs) were collected with a narrow-bore Pasteur pipette from the mono-ovular (Figure 1A,B) and poly-ovular antral follicles (Figure 1C,D). COCs were grouped separately according to estrus phase, ovarian follicle size—small antral (~0.2–0.39 mm), medium antral (~0.4–5.9 mm), and large antral (~6–10 mm)—and follicle type (mono-ovular or poly-ovular). The diameter of each individual follicle was measured using a graticule using a stereomicroscope during the release of the intra-follicular contents.

### 2.2. In Vitro Maturation and Meiotic Evaluation

COCs from each follicular size retrieved from poly-ovular and mono-ovular follicles were selected for IVM according to oocyte diameter (>100 mm), measured with a micrometer disc (Bausch and Lomb, USA); uniform ooplasm; and a compact cumulus cell mass surrounding the oocyte, as previously described [18]. The experiments were performed in six replicates. COCs from poly-ovular and mono-ovular follicles were incubated in TCM-199 supplemented with 10% fetal calf serum (Sigma #A6003-5G), 10 IU/mL human chorionic gonadotropin (Sigma #CG10-1VL), 0.25 mM pyruvate, 100 IU/mL penicillin (Sigma #K0521), 20 mg/mL streptomycin (Sigma #S-9137), and 2 μg/mL of ß-estradiol (E2) (E8875- 1G. Merck KGaA) at 38 °C and in a humidified atmosphere of 5% CO_2_ for 72 h as previously described [18,19]. In each replicate, the COCs of each follicle type were separately placed into 100 μL culture drops, each containing five to eight COCs, covered with mineral oil (Sigma #M8410-1L).

Since dog oocyte maturation is influenced by in vitro culture conditions, the same conditions were used for all oocytes subjected to IVM.

### 2.3. Assessment of Oocyte Maturation

After IVM culturing, the COCs were removed from the medium and placed into disposable dishes with PBS at room temperature (21–22 °C). With a fine-tipped pipette, the oocytes were denuded of the cumulus cells by gentle pipetting under a stereoscopic microscope (Krüss Optronic GmNH, Germany). Denuded oocytes were fixed in 100 μL of paraformaldehyde solution (4%) for 20 min and washed twice in PBS for 5 min each. Afterward, the oocytes were mounted on slides and incubated in 1 μg/L 4′-6-diamidino-2-phenylindole solution (DAPI, Thermo Fisher Scientific Inc., Rockford, IL, USA) for 5 min and washed twice in PBS solution for 5 min each to remove excess staining [18]. The oocytes were plated in a fluorescence mounting medium (Vectashield^®^; Vector Laboratories Inc., Burlingame, CA, USA) sandwiched between paraffin strings covered with a cover slip. The meiotic development of each oocyte was evaluated using an inverted epifluorescence Olympus IX71 microscope (UV emission 480 nm) equipped with an IX2-RFA lamp and a ProgRes-Capture Pro camera (Olympus, Tokyo, Japan).

**Figure 2:**
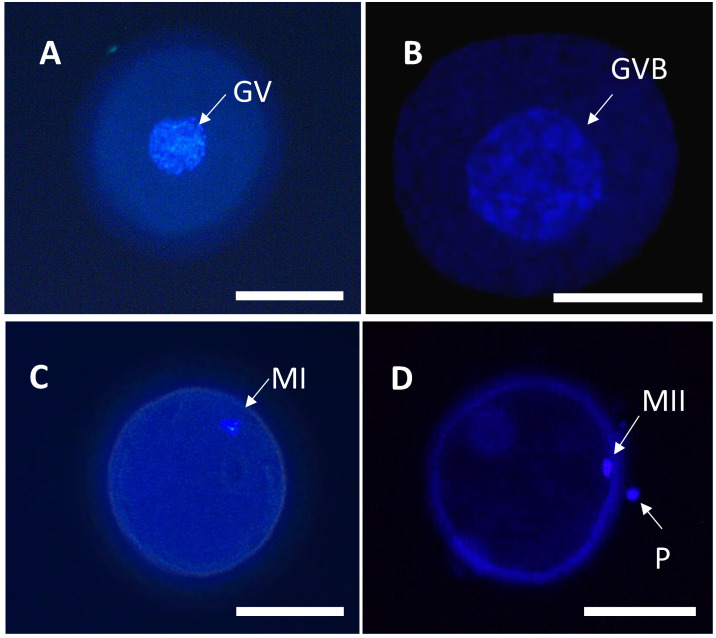
Meiotic stages in canine oocytes (**A**) Germinal vesicle (GV) immature or when the vesicle was clearly visible; (**B**) Germinal vesicle breakdown (GVBD) or resumption of meiosis; (**C**) First metaphase (MI) when chromosomes were condensed and present in equatorial view; (**D**) Second metaphase (MII) manifested by the presence of chromosomes in the second metaphase plate, with extrusion of the first polar body (PB). Samples were stained with 4′-6-diamidino-2-phenylindole (DAPI) and observed under an epifluorescence inverted microscope. Bar = 100 μm, 200× magnification.

Meiotic stages were classified into germinal vesicle (GV), germinal vesicle breakdown (GVBD), and first (MI) and second (MII) metaphases, as previously described [19] (Figure 2). Oocytes with no distinct nuclear structures, irregular chromatin distribution, or abnormal chromatin were excluded from the study.

### 2.4. Data Analysis

For each assay, samples were divided into groups according to their reproductive status and follicle type. To establish whether there was a difference between the meiotic development of oocytes from different follicle types (poly-ovular or mono-ovular follicles) and ovarian phases, the data were arcsine transformed and evaluated by ANOVA, followed by Duncan’s test for determining the differences between each group.

The analyses were performed using the InfoStat program (professional version, 2018). The model included the main effects of follicle type, phase of the estrus cycle, and their interaction for each stage of nuclear development.

A *p* value less than 0.05 was considered statistically significant.

## 3. Results

**Figure 3:**
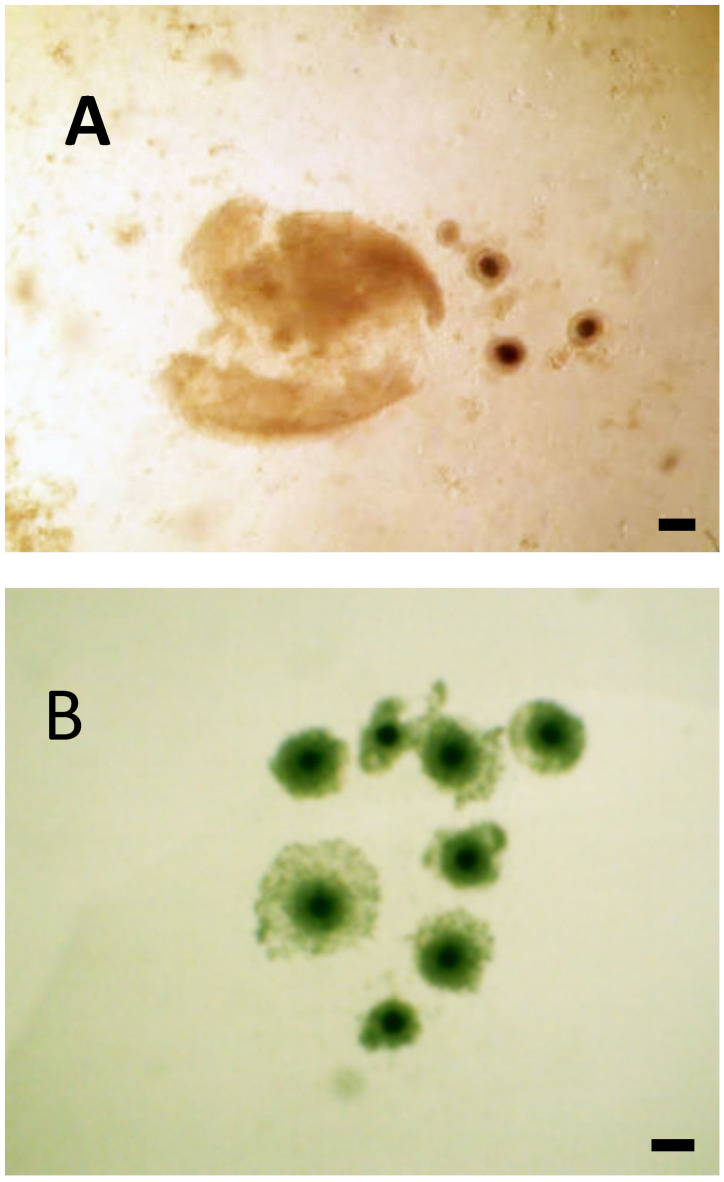
Representative photograph of oocytes from poly-ovular follicles (**A**) many oocytes emerging from a punctured poly-ovular follicle and (**B**) eight oocytes from poly-ovular follicle placed in the culture dish for selection, different sizes and characteristics of the oocytes are observed. Bar = 100 μm, 40× magnification.

Poly-ovular follicles were present in all the examined ovaries; however, mono-ovular follicles were the predominant type found in the ovaries at different sizes and phases of the estrus cycle. The number of oocytes per poly-ovular follicle ranged from two to eight. However, most contained approximately three to four oocytes per follicle. Some morphological differences were observed in the oocytes from the same follicle, including oocyte size (50 μm to >100 μm), cytoplasm darkness, and integrity of the cumulus cells (Figure 3A,B). As the number of oocytes within the same follicle increased, the difference between each oocyte also increased. Most oocytes from poly-ovular follicles containing two oocytes had similar sizes and appearances.

The meiotic development of oocytes from mono-ovular and poly-ovular follicles throughout the different phases of the estrus cycle is presented in Table 1. As follicular development progressed from small to medium or large size, the percentage of meiotic development increased in oocytes from both poly-ovular and mono-ovular follicles. During anestrus, most oocytes (57.1%) from small antral poly-ovular follicles remained arrested in the GV stage during the anestrus period and did not reach the MII stage, which was significantly different from those from mono-ovular follicles; however, few oocytes (3.2%) from small antral mono-ovular follicles during anestrus reached the MII stage. Oocytes from poly-ovular small antral follicles resumed meiosis (GVBD stage) at a lower rate (*p* < 0.05) than oocytes from mono-ovular follicles (24.3% and 34.1%, respectively) and reached a minor percentage of the MI stage compared to oocytes from mono-ovular follicles of the same size during anestrus (18.6% and 34.8%, respectively). The rate of oocytes arrested at the GV stage from medium poly-ovular follicles (33.3%) decreased at anestrus (*p* < 0.05) compared to those from small follicles. This rate of GV was higher (*p* < 0.05) than that of oocytes from mono-ovular follicles (20.9%), but without differences in the MII stage between both types of follicles (10.3% and 9.1%, respectively).

At proestrus, there were differences (*p* < 0.05) in the percentage of oocytes arrested at the GV stage between oocytes from mono- and poly-ovular medium antral follicles (36.8% and 20.3%, respectively), increasing (*p* < 0.05) final maturation with size. During estrus, oocytes from medium and large poly-ovular follicles showed significant differences in the GV stage (*p* < 0.05). The lowest rate (*p* < 0.05) of oocytes arrested at the GV stage was observed in oocytes from medium (8%) or large (7.6%) mono-ovular follicles at the estrus phase, while the highest rates of oocytes at the MII stage were those from mono-ovular and poly-ovular follicles of medium (21.9% and 26.2%, respectively) and large (20.7% and 22.2%, respectively) sizes, without differences (*p* > 0.05) in the final meiotic status (MII) between oocytes from mono-ovular or poly-ovular follicles.

The diestrus phase showed differences (*p* < 0.05) between oocytes in poly- and mono-ovular follicles arrested at the GV stage in small (31.3% and 20.9%, respectively) and medium (27.8% and 18.6%, respectively) antral follicles. Although more (*p* < 0.05) oocytes from mono-ovular follicles reached the MI stage in comparison to oocytes from poly-ovular follicles, oocytes from both types of follicles did not differ in reaching the final maturation stage at MII in small (8.0% and 7.1%) or medium (6.0 and 6.1%) follicles.

## 4. Discussion

Despite the high incidence of poly-ovular follicles in dogs, this is the first report describing the ability of canine oocytes derived from poly-ovular follicles to mature in vitro. In this study, the poly-ovular follicles were found in all stages and phases of the estrous cycle. The incidence of mono-ovular follicles was predominant in agreement with other species [1,3,20]. However, the presence of poly-ovular follicles was higher than that described in most mammals [10,20].

In contrast to other mammals, the development of ovarian follicles in dogs begins after birth, and although no clear evidence shows that follicular development occurs in a wave-like pattern during each estrous cycle, the follicles begin to grow in the anestrus period when the circulating follicle-stimulating hormone (FSH) increases [21], which is a characteristic event of follicular recruitment in other species [22,23]. In anestrus, there were mainly small follicles growing to medium-sized follicles. After 72 h of IVM, the oocytes from small antral follicles—both poly-ovular and mono-ovular follicles—showed the lowest meiotic competence compared to oocytes from other sizes or reproductive phases. It has been reported that oocytes recovered from anestrus ovaries are unable to survive or resume meiosis [24]. In our study, even though many of the oocytes retrieved from these follicles and submitted to IVM remained arrested in the prophase of meiosis, a few of them were able to resume meiosis (GVBD stage) in either mono-ovular or poly-ovular follicles; however, no oocytes from small poly-ovular follicles were able to reach the final maturation state at MII. Intrafollicular requirements at the beginning of development are possibly more critical when there is more than one oocyte inside. In a more advanced stage of follicular development still in anestrus, some oocytes derived from medium poly-ovular follicles could reach the MII stage in vitro at a similar percentage as those derived from mono-ovular follicles. The follicle size, associated with the level of follicular development, is related to the quality of immature oocytes since the maturation of oocytes from small to medium or large follicles may depend on different intrafollicular requirements, implicating messenger RNA or protein stores as factors involved in oocyte meiotic competence [25]. In vivo, the acquisition of meiotic competence occurs progressively during follicular growth. Therefore, many oocytes isolated at an early stage of follicular development may fail to progress through the MII stage under culture conditions. Similarly, in cattle, the size of follicles affects oocyte competence [26,27].

During proestrus, a direct relationship between follicular size and oocyte competence was also observed after IVM in poly-ovular follicles, with the oocytes harvested from small follicles being less competent in reaching the MII stage than those from medium follicles. This difference was not observed in oocytes from mono-ovular follicles. In other species, there is some retardation in the developmental capacity of poly-ovular follicles in acquiring the ability to grow to more advanced stages [28]. The minor competence in oocytes derived from small follicles during the anestrus and proestrus phases compared to the competence in medium follicles seems to be more critical in oocytes from poly-ovular follicles than in those from mono-ovular follicles.

The gene expression patterns of the oocyte and follicular microenvironment in which the oocyte grows and matures are factors that contribute to oocyte quality and meiotic competence [29]. Changes in the level of receptor mRNAs of steroids, growth factors, and gonadotropins [17,30] in oocytes and follicular cells may reflect the differences among oocytes from follicles of different sizes. Critical roles for oocytes in folliculogenesis and the crosstalk between oocytes and granulosa cells have been established [31]. Therefore, endocrine, and metabolic signals that regulate follicular growth and influence oocyte development can be directly affected by the number of oocytes inside each follicle. Accordingly, the intrafollicular concentration of steroids depends on the number of oocytes in each follicle [28]. Steroid production is regulated by intrafollicular growth factors that modulate the actions of gonadotropins on granulosa and theca cells [13,32], either through changes in hormone levels in the follicular fluid or via granulosa–oocyte interaction [33]. In bovines, the highest concentrations of transforming growth factor (TGF)-members have been found in the follicular fluid of the smallest follicles sampled at the beginning of the follicular wave [34]. In previous studies of canines, we found that members of the TGF superfamily, GDF-9 and BMP-15, were highly expressed during the early stages of follicular growth, mainly during the anestrus phase [15,16]. In addition, mice lacking BMP-15 or GDF-9 have more poly-ovular follicles than wild-type mice [12,35]. These findings suggest that intrafollicular growth factors could be required at the beginning of follicular growth to provide steroidogenic capacity to the follicular cells and the maturation competence of oocytes. Hence, lower levels of growth factors such as GDF-9, and BMP-15 in early follicles, could enhance the formation of poly-ovular follicles due to the influence of these factors on the nests’ breakdown [9]. On the other hand, a higher number of oocytes within the follicle would make them compete with one another, diminishing their capabilities. Similarly, we found that as the number of oocytes within a single follicle increased, the differences among the oocytes also increased. In fact, oocytes collected from the same follicle are often at different stages of maturity [13] and possibly at different stages of the cell cycle.

The highest rate of oocytes at the MII stage was observed in the estrus phase in both poly-ovular and mono-ovular follicles. Although the percentage of oocytes arrested at the GV stage and those that reached the MI stage in the estrus phase were different in oocytes from mono-ovular and poly-ovular follicles, the ability to reach the final stage of maturation at MII did not differ. In addition, no differences were found in the final meiotic maturation stage between both follicular types during the diestrus period. In dogs, the estrus and diestrus phases are preceded by a surge in luteinizing hormone (LH) which initiates many biochemical processes in the ovary, promoting an acute increase in progesterone levels [21]. The progesterone concentration in the follicular fluid recovered from follicles during the estrus phase is considerably higher than that in follicles obtained from proestrus bitches [36,37]. The significant increase in progesterone levels after the peak of LH might influence the final maturation of canine oocytes, matching the maturation capacities of oocytes from poly-ovular follicles since progesterone is a crucial element in the oocyte maturation cascade. Several studies have correlated oocyte maturity with progesterone levels [38,39].

During diestrus, the rate of meiotic development decreased compared to that during the estrus phase, and there were no differences in MII percentage in oocytes from either poly-ovular or mono-ovular follicles. In diestrus, the predominant structures in the canine ovaries are CLs, and according to a previous study in bovines, the presence of a CL in the ovary exerts a negative effect on oocyte maturation because the CL receives the highest rate of blood flow compared to other ovarian cells [40].

The effect of the estrus cycle on the in vitro meiotic maturation of canine oocytes has been described in other reports, with controversial results. According to the data reported in a meta-analysis approach [41], more oocytes from the proestrus, estrus, and luteal phases resumed meiosis than those from the anestrus phase. Little is known about the factors that regulate oocyte maturation in vivo in this species, and there are no studies regarding the developmental capacity of oocytes from poly-ovular follicles in dogs. Our study demonstrated differences in oocytes from poly-ovular and mono-ovular follicles according to the estrous phase and follicle size, possibly due to changes in the follicular cells and microenvironment inside each follicle. Although we gathered only healthy follicles and the same morphological selection of oocytes was made in all experiments from poly-ovular and mono-ovular follicles, the oocytes retrieved from those follicles resulted in different meiotic timing and competence depending on follicle sizes and the ovarian cycle. Therefore, these factors should be considered, as they are important for the variability of canine oocytes matured in vitro.

## 5. Conclusions

This study demonstrated for the first time in canines that although oocytes from poly-ovular follicles can resume meiosis at a slower rate than those from mono-ovular follicles, they can nevertheless grow, and in vitro maturation of such oocytes is possible. Furthermore, the relationship between the maturation capacity of oocytes from poly-ovular and mono-ovular follicles depends on the ovarian cycle and follicular size. Understanding the oocyte development process in each type of follicle will collaborate for improving IVM protocols in this species, a prerequisite for further advancements in the field of reproductive biotechnology.

## Figures and Tables

**Table 1:** Meiotic development of oocytes from poly-ovular (*p*) and mono-ovular (M) follicles throughout the estrus cycle in dogs.

Phase of the Estrous Cycle		Follicles		Meiotic Development									
				GV (%)		GVBD (%)		MI (%)		MII (%)		Total Oocytes (n)	
			Type	P	M	P	M	P	M	P	M	P	M
		Size											
Anestrus	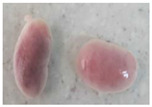	SA		57.1 ^ax^	31.0 ^ay^	24.3 ^ax^	34.1 ^ay^	18.6 ^ax^	34.8 ^ay^	0.0 ^ax^	3.2 ^ay^	116	138
		MA		33.3 ^bx^	20.9 ^bx^	28.6 ^ax^	26.8 ^ax^	23.8 ^ax^	42.2 ^ay^	10.3 ^bx^	9.1 ^bx^	104	127
											
Proestrus	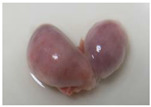	SA		33.3 ^bx^	32.2 ^ax^	30.3 ^ax^	23.3 ^ax^	29.3 ^ax^	48.3 ^ay^	6.1 ^ax^	15.0 ^cy^	109	132
		MA		36.8 ^bx^	20.3 ^by^	26.3 ^ax^	21.4 ^ax^	24.6 ^ax^	40.2 ^ay^	12.3 ^bx^	16.1 ^cx^	90	113
											
Estrus	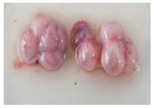	SA		20.5 ^bx^	8.o ^cy^	23.1 ^ax^	21.7 ^ax^	34.5 ^ax^	50.4 ^ay^	21.9 ^cx^	26.2 ^cx^	110	123
		MA		33.3 ^cx^	7.6 ^cy^	35.0 ^bx^	17.7 ^ay^	33.0 ^ax^	53.5 ^ay^	20.7 ^cx^	22.2 ^cx^	99	119
											
Diestrus	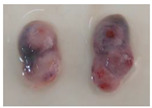	SA		31.3 ^bx^	20.9 ^by^	38.6 ^abx^	29.4 ^ay^	22.1 ^ax^	40.0 ^ay^	8.0 ^abx^	7.1 ^bx^	128	131
		MA		27.8 ^bx^	18.6 ^by^	31.6 ^abx^	36.5 ^ax^	30.6 ^ax^	42.8 ^ay^	6.0 ^abx^	6.1 ^bx^	116	123
											

Meiotic stages: GV, germinal vesicle; GVBD, germinal vesicle breakdown; MI, first metaphase; MII, second metaphase. Follicle type: SA, small antral ~0.2–0.39 mm; MA, medium antral ~0.4–5.9 mm; LA, large antral ~6–10 mm (only observed in estrus phase). Representative photos of ovaries are shown at each stage of the estrous cycle. ^a–c^ Within a column and meiotic stage, numbers with different superscripts are different (*p* < 0.05). ^x,y^ Within a row and meiotic stage, numbers with different superscripts are different (*p* < 0.05).

## Data Availability

The data of this study are available on request from the corresponding author.
